# Stress in captive Blue-fronted parrots (*Amazona aestiva*): the animalists’ tale

**DOI:** 10.1093/conphys/coz097

**Published:** 2019-12-08

**Authors:** Alan Chesna Vidal, Mar Roldan, Maurício Durante Christofoletti, Yuki Tanaka, David Javier Galindo, José Maurício Barbanti Duarte

**Affiliations:** São Paulo State University (UNESP), School of Agricultural and Veterinarian Sciences, Deer Research and Conservation Center (NUPECCE), Jaboticabal, São Paulo 14884-900, Brazil

**Keywords:** Psittacidae, cortisol, captivity, corticosterone, urofaecal glucocorticoid metabolites

## Abstract

Understanding stress physiology is crucial for species management because high levels of stress can reduce reproduction and the individual’s ability to face threats to survive. One of the most popular methods for non-invasive monitoring of animal endocrine status is the glucocorticoid (GC) metabolite measurements, which can provide important information about how animals are affected by their surrounding environment. Here, we carried out the biological validation of corticosterone enzyme immunoassays (EIAs), which together with a cortisol EIA was used to quantified the concentrations of urofaecal GC metabolites (uGCMs) in wild and captive Blue-fronted amazon parrots (*Amazona aestiva*). Urofaecal GC concentrations were significantly higher (*P* < 0.05) in free-living parrots (157.9 ± 18.5 ng cortisol/g and 61.14 ± 23.5 ng corticosterone/g dry urofaecal sample) than in those kept in captivity, which showed the comparable levels of GC metabolites independently of the management system applied. The higher uGCM levels obtained in the wild population point to an adaptive response for survival and species propagation in a more challenging environment, in comparison with captive animals. Furthermore, the lower uGCM concentrations in captive parrots may indicate an adaptive capacity of the species *A. aestiva* to captivity and its potential as a legal pet. The corticosterone EIA applied in this study proved to be an effective technique for the adrenocortical activity monitoring in this species. We discuss our findings considering the management and destiny given to wild-caught birds that are kept in confinement or returned to nature.

## Teaser

Lay summary

Captivity has traditionally been considered a stressful habitat for wildlife. In this study, urofaecal glucocorticoid metabolites of wild and captive Blue-fronted amazon parrots (*Amazona aestiva*) subjected to different husbandry systems were determined. Results reveal lower corticoid levels in individuals maintained in captivity than in the free-ranging ones.

## Introduction

The Psittacidae family represents one of the most challenging avian groups to be studied currently, since one-third of all known parrot species are considered at risk (Berkunsky *et al.*, 2017). Brazil is home to the largest number (87) of Psittacidae species in the world (Forshaw, 1989), although approximately half of these species are under a category of threat in the Red List of Threatened Species of the International Union for Conservation of Nature and Natural Resources (IUCN) (IUCN, 2018). Among Brazilian parrots, the Blue-fronted amazon (*Amazona aestiva*), also known as Blue-fronted parrot, Turquoise-fronted amazon or Turquoise-fronted parrot, is of particular interest for being one of the most popular parrot species kept in captivity (Seixas and Mourão, 2002). It has a wide range of distribution, from northeast Brazil to Bolivia, Paraguay and northern Argentina, and a large variety of habitats, mainly forested and Cerrado areas (Juniper and Parr, 1998), although they can also be found practically throughout the country flying in flocks or couples.

Despite its current classification as Least Concern by the IUCN, this is one of the most common illegal traded parrots in Brazil and other Latin American countries (Pires, 2012). It is widely prized as a pet because of its sociability, intelligence, striking plumage and ability to copy different sounds and imitate the human voice (Tella and Hiraldo, 2014). Accurate data on the annual number of parrots taken from their natural habitat to supply the illegal pet trade are unknown, but the extensive trade pressure has justified its inclusion in the ‘National Action Plan for the Conservation of Threatened Parrots of the Atlantic Forest’ in Brazil (Lopes *et al.*, 2018). Chick poaching from nests for the pet trade and habitat destruction by direct human activities are causing an alarming decline in this and other Amazona species populations (Juniper and Parr, 1998; Wright *et al.*, 2001; Berkunsky *et al.*, 2017). In Argentina, where the practice of removing wild animals from nature in quotas was legal, the number of Blue-fronted parrots authorized for export as part of the pet trade by Argentinian provinces between 1983 and 1991 was over 360 000 individuals (Beissinger and Bucher, 1992), revealing the strong presence of this species as a pet. In Brazil, besides private households, the Blue-fronted amazon is also very frequently found in zoos and in rescue government centres such as Centres of Rehabilitation of Wild Animals or Wild Animals Selection Centres. In addition, these birds are among the most common species kept in captivity in this country (Kuhnen *et al.*, 2012), although their adaptation to the artificial habitats does not always happen. Unlike dogs and cats, which have been under human selection for thousands of years to become permanent pet companions, parrots are only in the very early stages of domestication and are genetically comparable to their wild ancestors, presenting, therefore, similar needs in captivity (Meehan *et al.*, 2003; Williams *et al.*, 2017). In this sense, when basic requirements are not provided, captive psittacines have a high predisposition to develop behavioural disorders associated with chronic stress (Ferreira *et al.*, 2015).

In birds, like in mammals, the secretion of adrenal-derived glucocorticoid (GC) hormones is an important mechanism for dealing with stressors so that they are widely used as biomarkers to examine welfare and the adrenocortical status in a variety of taxa (Millspaugh and Washburn, 2004). Whereas cortisol is the major endogenous GC released in fish and most mammals, corticosterone is primarily secreted in birds, reptiles, amphibians and some rodents (Romero, 2004). These stress hormones are physiologically secreted at relatively low levels in undisturbed animals (baseline thereafter), fluctuating with circadian and seasonal rhythms in accordance with predictable metabolic demands to maintain the animal homeostasis (Reeder and Kramer, 2005). Although an acutely short-term secretion in response to unexpected perturbations in the environment, such as storms or predation, allows animals to cope with emergency situations, elevated GC levels over a prolonged period (chronic stress) may negatively influence their fitness, even leading to failure in reproductive function (Landys *et al.*, 2006; Thierry *et al.*, 2013). Unfortunately, it is currently difficult to differentiate whether elevated GC levels are secreted in response to unhealthy chronic stress or a healthy coping response. Knowing the baseline GC levels is, in many cases, the only way to answer important questions about stress physiology in wild birds, but this approach presents some limitations. For example, obtaining adequate and repeated blood samples in wild and/or small animals, such as many bird species, is particularly challenging, and the procedure itself may rise doubts about the interpretation of the adrenocortical responses because GC levels can increase in response to the handling stress (Touma and Palme, 2005; Palme, 2019). In fact, researchers have considered that only samples collected in less than 3 min after capture will allow for accurate measurements of the baseline GC concentrations (Romero and Reed, 2005). To tackle all these drawbacks, non-invasive methods have been extensively developed in the last decades, being the measurement of faecal GC metabolites one of the most used procedures for stress assessment in domestic and wild vertebrates (Millspaugh and Washburn, 2004). In parrots, different studies have also applied such methods to non-invasively monitor increased adrenocortical activity in different species (e.g. Owen and Lane, 2006; Ferreira *et al.*, 2015; Costa *et al.*, 2016). And, although only some of them use fully validated methods, this approach seems to offer a very attractive sampling alternative for this group of birds (Young and Hallford, 2013; Ferreira *et al.*, 2015; de Almeida *et al.*, 2018). Understanding how the physiology of parrot changes with their environment becomes crucial for their conservation. Therefore, we aimed at investigating the influence of different management systems on the urofaecal glucocorticoid metabolites (uGCM) excretion of free-living and captive individuals of *A. aestiva*. Our work will also promote a discussion about the need to return captive animals to nature and encourage more studies directed to the welfare of species in captivity.

## Material and methods

### Animal study and sample collection

This study was approved by the Animal Ethics and Welfare Committee (Comitê de Ética e Bem-estar Animal) of the School of Agricultural and Veterinarian Sciences (Faculdade de Ciências Agrárias e Veterinárias, FCAV) UNESP, Jaboticabal, SP, Brazil (protocol number 009350/11).

**Figure 1:**
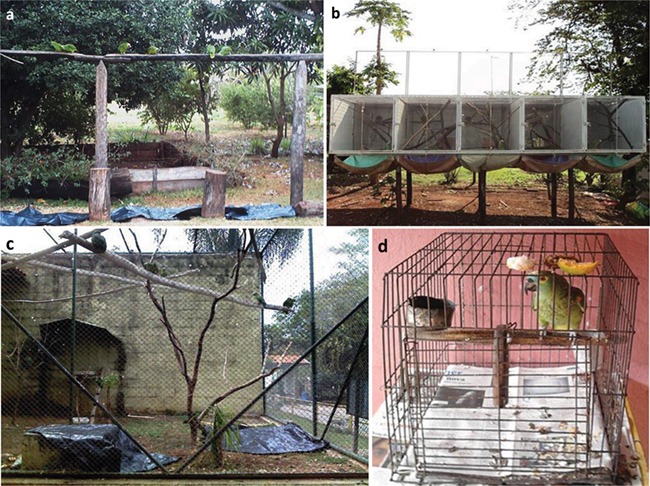
The husbandry systems used in the study. (**a**) Free-living Blue-fronted parrots in collective feeders in the Fazenda São Francisco, Pantanal region; (**b**) pair-housed parrots in suspended breeding aviaries at the Brisa commercial breeder; (**c**) Blue-fronted parrot enclosure at the Municipal Zoo of Piracicaba (São Paulo) with the plastic tarpaulins; (**d**) example of an *A. aestiva* individual living as pets in a private house.

In avian species, faeces and urine are mixed in the cloaca and both portions are usually excreted together in form of droppings (Möstl *et al.*, 2005). Although in some occasions it is possible to separate these two fractions, using them together as a single sample has been recommended to achieve a more integrated estimate of the total hormones (Millspaugh and Washburn, 2004). Therefore, we collected complete droppings (also referred as urofaecal samples) of 86 Blue-fronted parrots of different origins (wild, commercial breeder, zoo and pets) in Brazil between July and August 2011 (see Fig. 1). Most samples were collected between 12:00 and 18:00 p.m. for all groups, thereby attenuating any circadian influence in hormone levels. All droppings were placed in individually labelled plastic microtubes and kept frozen at −20°C until further analysis.

#### Wild animals

Urofaecal samples of 24 animals from a free-living population of the Pantanal region in the State of Mato Grosso do Sul, specifically from the Fazenda San Francisco, were collected from collective feeders set for tourist observation of different psitacid species. Defecations were observed from a distance not to disturb the animals, and droppings were collected immediately after defecation to avoid sample mixing, in plastic sheets previously placed under the feeders.

#### Commercial breeder animals

Ten males and 10 females of Blue-fronted parrots from the Brisa commercial breeder, located in Jaboticabal, State of São Paulo, Southeaster Brazil (21°16′46″S, 48°1343″W; Licenses IBAMA CTF: 263703, AM: 00024/2008-SP and SMA AM:118748/2015), were sampled. Adult animals (≥4 years old) were pair-housed in suspended breeding aviaries (1 m high × 1 m wide × 2 m deep) with visual sidebars, equipped with external vertical wooden nest boxes (45 cm high × 20 cm wide × 20 cm deep) and located one meter above the ground. Some animals came from rescue centres (i.e. seized from illegal parrot traders) and others were born at the breeder, but all of them were in captivity for years. Males and females were separated by a net partition to facilitate the identification of the samples in each cage, and all droppings accumulated during the time previously predefined were collected from a plastic tarpaulin extended on the floor, considering them as an only sample.

#### Zoo animals

Urofaecal samples of 22 individuals were collected at the Municipal Zoo of Piracicaba, in the State of São Paulo. All animals were housed in a collective enclosure and came to the zoo as chicks, being in confinement for a longer period prior to the onset of the study. Samples were collected as described for the wild animals, except that the plastic tarpaulins were placed underneath two of the main perches in the enclosure.

#### Pets

Twenty parrots living as pets in individual cages in family houses were used. Type of feed and handling were documented to identify possible variations among these samples (Table 1). The urofaecal samples were collected 1 h after the cages were cleaned, and these were considered as a single sample.

**Table 1:** Information about the Blue-fronted parrots’ management maintained as pets in private houses (Jaboticabal/SP)

Individual	Age (year)^a^	Handling	Feeding
1	1	Animal contained within the cage only at night	Ration, fruits
2	30	Animal contained within the cage only at night	Sunflower seeds, rice, cookies, fruits, potatoes
3	10	Animal contained within the cage only at night	Sunflower seeds, rice, cookies, fruits, potatoes
4	15	Animal contained within the cage only at night	Fruits, sunflower seeds, bread with coffee, lettuce
5	8	Animal contained within the cage only at night	Seed mix, fruits, bread with coffee, cookies
6	6	Animal contained within the cage only at night	Seed mix, fruits, bread with coffee, cookies
7	20	Animal contained within the cage only at night	Sunflower seeds, corn, peanuts, fruits
8	12	Animal on the loose all day	Fruits, seed mix
9	2	Animal contained within the cage only at night	Sunflower seeds, apple, bread
10	1	Animal contained within the cage only at night	Sunflower seeds, cornmeal, cookies, fruits
11	1	Animal held in the cage most of the day	Ration, Sunflower seeds, seed mix, fruits
12	5	Animal held in the cage most of the day	Sunflower seeds, fruits, popcorn, bread
13	5	Animal held in the cage most of the day	Sunflower seeds, fruits, popcorn, bread
14	30	Animal contained within the cage only at night	Sunflower seeds, home-cooked food, bread
15	17	Animal held in the cage all day	Sunflower seeds, corn, fruits, vegetables
16	17	Animal held in the cage all day	Sunflower seeds, corn, fruits, vegetables
17	2	Animal contained within the cage only at night	Fruits, Sunflower seeds, cake, bread with milk
18	3	Animal held in the cage most of the day	Pelleted ration, fruits, sunflower seeds
19	^*^	Animal contained within the cage only at night	Fruits, seed mix, bread
20	^*^	Animal held in the cage most of the day	Sunflower seeds, fruits

a ^a^The age of parrots was considered as the period between the moment they arrived at the private house and the experiment time.

^*^Unknown age.

### Biological validation of the corticosterone enzyme immunoassay

**Figure 2:**
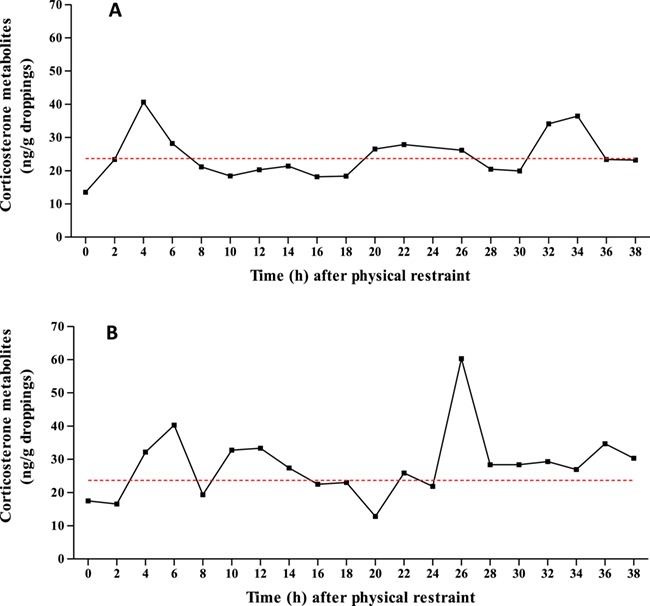
Urofaecal CM concentrations (ng/g dry matter) profile from a physical restraint procedure (h 0) in (**A**) a female and (**B**) a male Blue-fronted parrot (*A. aestiva*) using a corticosterone EIA. Dashed line represents the mean of pre-restraint uGCM concentration (ng/g dry matter).

The validation procedure was conducted on a male and a female maintained in individual cages in our research centre where they spent 30 days to adapt to their new environment before starting the biological validation procedure. The animals were physically immobilized and held in the hands for 30 min to provoke a stressful stimulus. The urofaecal collection started just after the end of the restraint period (time 0), and it continued at 2-h intervals for a period of 38 h. Droppings from both individuals were available at all collection intervals, except at 24 h in the case of the female, and presented no abnormal characteristics during all the experiment. The samples were treated in the same way as those from the other groups. Some urofaecal samples were also collected prior to the validation procedure to establish natural concentrations of GCM. The corticosterone enzyme immunoassay (EIA) successfully detected the expected increase in corticosterone metabolites (CMs) following the physical restraint (Fig. 2). Such a procedure caused a very comparable adrenocortical response in both sexes achieving values of 40 ng/g in the male after 6 h and 41 ng/g in the females after 4 h. A second CM rise can be observed after 24 and 30 h in the male and female, respectively.

### Extraction of uGCM

Urofaecal samples were first dried in an oven (Mod. 320-SE, Fanem Ltda., São Paulo, Brazil) at 56°C for ~72 h according to the method previously described by Hamasaki *et al.* (2001). The extraction procedure was based on the protocol described previously by Touma *et al.* (2003). Briefly, 1 ml of 80% methanol was added to 0.05 g (±0.002) of pulverized samples placed into 5 ml centrifuge tubes, and the mixture was agitated in a vortex for 30 min. Samples weighting less than 0.05 g received a proportional volume of methanol, and those weighing less than 0.01 g were excluded from the analysis. The samples were then shaken for 12 h on a mechanical shaker (Mod. AP22, Phoenix Ltda, Araraquara, Brazil), and the extracts were subjected to ‘centrifugation’ at 1500 rpm for 20 min, with the resulting supernatant stored in a −20°C freezer until further analysis.

### Enzyme immunoassays

Urofaecal cortisol concentrations were determined using a previously validated cortisol EIA for this species (Fujihara *et al.*, 2014). Polyclonal cortisol antiserum (R4866) and horseradish peroxidase (HRP) ligands were supplied by Dra. Coralie Munro (Clinical Endocrinology Laboratory, University of California, Davis, CA, USA) and used at 1:8500 and 1:20 000 dilutions, respectively, with cortisol standards varying from 3.9 to 1000 pg/well. The antiserum presented the following cross-reactivities (provided by the laboratory): 100% cortisol, 58.3% prednisolone, 10.9% prednisone, 7.0% cortisone, 5.7% 11-deoxycortisol, 1.9% 21-deoxycortisol, 0.9% 17a-hydroxyprogesterone, 0.9% dexamethasone, 0.4% triamcinolone and <0.004% corticosterone, progesterone and DHEA. Urofaecal corticosterone levels were determined using the same EIA for cortisol with some modifications: 33.3 μl of corticosterone CJM06 antibody (also supplied by Dra. C. Munro) were initially pipetted onto micro plates for incubation, and the polyclonal anticorticosterone antiserum and the HRP conjugated corticosterone label were diluted to 1:15 000. Cross-reactivity of the CJM06 anti-corticosterone antiserum was reported as 100% with corticosterone, 14.25% with desoxycorticosterone and 0.9% with tetrahydrocorticosterone (provided by the laboratory).

Immunoassays were validated for Blue-fronted parrot droppings by demonstrating parallelism between serial dilutions of urofaecal extracts (diluted to 1:2–1:2048) and the respective standard curve (20 ng/ml). The sensitivity of the assays was calculated as the value 2 SD from the mean response of the zero (Bo) tube, being 0.60 ng/g (*n* = 15) and 1.96 ng/g (*n* = 10) for cortisol EIA and corticosterone EIA, respectively. Inter-assay coefficients of variation (CV) for low (~30%) and high (~70%) value quality controls were 3.1 and 7.4% for the corticosterone EIA and 11.8 and 15.7% for the cortisol EIA, respectively. All samples were re-analysed if the duplicate CV was >10%; therefore, intra-assay CV were <10% for both EIA. All urofaecal GC concentrations were expressed as ng/g dry matter.

### Data analysis

The data sets were presented as mean ± SEM and were analysed using GraphPad Prism software version 6.0 (GraphPad Prism Statistical Software, Inc., CA, USA). The uGCM data were normally distributed and were ln-transformed prior to analysis to normalize errors and graphical representation. A one-way analysis of variance with Tukey’s Multiple Comparison (Post hoc) Test was performed to compute simple associations and comparisons between the different parameters and the four experimental groups. Differences of *P* < 0.05 were taken as significant for all statistical tests.

## Results

**Figure 3:**
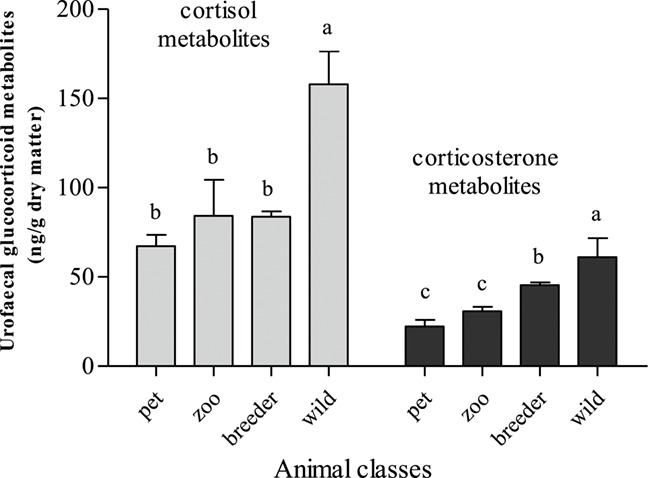
Concentrations (ng/g dry matter) of uGCM in 86 Blue-fronted parrots (*A. aestiva*) subjected to four different husbandry systems: animals held as a pet in private houses (*n* = 20), under captivity in a zoo (*n* = 22), from a commercial breeder (*n* = 20) and wild animals (*n* = 24). Gray bars: mean ± standard error of the mean urofaecal cortisol metabolite concentrations. Black bars: mean ± standard error of the mean urofaecal CM concentrations. Different letters indicate significant differences (*P* < 0.05).

uGCM concentrations of free-ranging and captive Blue-fronted parrots under different management systems were compared and the results are shown in Fig. 3. GC levels in droppings from wild parrots ranged from 61- to 303-ng/g urofaecal sample, with a mean of 174 ng/g for cortisol, and from 33- to 87-ng/g urofaecal sample, with a mean of 61 ng/g for corticosterone. In captive parrots, urofaecal cortisol and CM concentrations varied, respectively, between 31 and 112, and 11- and 54-ng/g urofaecal sample, with means of 72- and 33-ng/g urofaecal sample. Free-living parrots presented significantly higher uGCM concentrations in comparison with those kept in captivity. In addition, all captive groups showed comparable levels of urofaecal cortisol metabolites independently of the management system applied, whereas CM levels were slightly but significantly higher for the parrots from the commercial breeder, compared with the animals kept in the zoo or living as pets. These results point to a marked effect (*P* < 0.05) of the environment on the adrenal activity.

## Discussion

We aimed to investigate the influence of different management systems on excreted GC levels from wild and captive Blue-fronted parrots using a cortisol and corticosterone EIA. Results of the physical restraint showed that the corticosterone EIA can be used for quantifying CM in urofaecal samples of Blue-fronted amazon and, consequently, for monitoring adrenocortical response in captive and free-ranging individuals. Moreover, different studies have already demonstrated the possibility of using other validated EIA to evaluate the hypothalamic-pituitary-adrenal (HPA) axis in this species (Fujihara *et al.*, 2014; Ferreira *et al.*, 2015). As shown in Fig. 2, the restraint was stressful enough to provoke urofaecal CM peaks. The first peaks appeared only a few hours after the procedure in both sexes, between 4 and 6 h, in agreement with the reported gut-passage delay times (2.1–5.5 h) in Amazon parrots (McMillan, 1994). In this same species, Ferreira *et al.* (2015) obtained similar results using a cortisone assay reporting peaks 3–9 h after stimulation by Adrenocorticotropic hormone (ACTH) challenge in both sexes, whereas Fujihara *et al.* (2014), using a cortisol assay, obtained more diverse results with peaks between 2 and 4 h after the ACTH administration for males and 10 h after in females. Furthermore, these results are comparable with those reported by de Almeida *et al.* (2018) in blue-and-yellow macaws (*Ara ararauna*) or by Sinhorini (2013) in golden parakeets (*Guaruba guarouba*), who reported a second peak 20 h and between 11 and 15 h after the Adrenocorticotropic hormone (ACTH) challenge, respectively.

Although non-invasive methods have already been used to measure stress response in captive Blue-fronted parrots (e.g. Fujihara *et al.*, 2014; Ferreira *et al.*, 2015; Queiroz *et al.*, 2016; Matos *et al.*, 2017), this is the first study to quantify GC concentrations in a wild population of this species. Significantly higher GC levels were obtained in free-living parrots, which may be associated with a more challenging and, consequently, a more energetically demanding living environments compared with those provided to captive parrots. Activities such as foraging, immune responses or thermoregulation entail a doubtless expenditure of energy that is reflected in a higher GC baseline (Goymann *et al.*, 2017; Jimeno *et al.*, 2017). Wild parrots, which spend a significant part of the day in foraging activities, flying even kilometres to increase their chances of finding food (van Zeeland *et al.*, 2013), are therefore supposed to have higher GC levels than those with more limited activity.

This study was carried out at the onset of the reproductive period of this species, a very energetically demanding period that typically results in an elevation of the GC basal levels (Romero, 2002). This energy demand, however, seems to be lower in animals under captivity compared with their wild counterparts, probably because it is associated with their lower foraging requirements (Speakman, 2005). These findings are in agreement with those reported by Gardiner and Hall (1997) who observed that reproductive free-living harbour seals (*Phoca vitulina*) presented higher cortisol levels than those kept in captivity, likely triggered by a stronger dispute for food and mates. Therefore, reproduction may be responsible, in part, for the higher stress hormone levels in free-ranging parrots. This is supported by the fact that CM concentrations in the commercial breeder parrots are increased when compared with other captive animals in the zoo or living as pets, which were not reproducing. However, such an increase in GC levels during reproduction can be considered normal and even necessary to increase the chances of reproductive success in birds (Pereira *et al.*, 2018). For example, in the breeding period, increased GC concentrations were essential for promoting the foraging activity to achieve a good body condition (Stearns, 1992; Descamps *et al.*, 2011; Hennin *et al.*, 2016) and for enhancing the parental reproductive effort (Romero, 2002; Love *et al.*, 2004; Thierry *et al.*, 2013). Although no additional data on stress hormone levels are available for this species in their natural environment, we can infer that these differences in GC values among wild and captive parrots are attributed to non-stressful adaptive strategies that allow animals to deal with unpredictable environmental disturbances (McEwen and Wingfield, 2003). However, even with a potential beneficial effect, increases in GC baseline have been traditionally considered as pernicious, placing these physiological responses at the same level as those that occur in reactions to unexpected and life-threatening stimuli, in which energetic demand increases and corticoids are acutely released (Scheuerlein *et al.*, 2001). The breeding success and the lack of abnormal behaviours and the population stability observed since 1997 (Seixas, 2009) in our free-living group seem to suggest that the higher uGCM concentrations obtained in this individuals are normal physiological levels for them.

Lack of food has been closely associated with higher GC secretion in wild birds (Kitaysky *et al.*, 1999, 2001; Lendvai *et al.*, 2014). In the case of the free-living Blue-fronted amazon parrots, the urofaecal samples were collected in July, at the onset of the dry season, when food availability is at its highest for these animals in the Pantanal region (Seixas, 2009). But, in addition to natural resources, these animals received an extra supplementation of food provided by the Fazenda San Francisco staff to bring these parrots closer to tourists, thus helping to meet part of their daily energy demand. This situation of abundant food is another reason why we hold the opinion that the uGCM levels obtained from this group of parrots in the Pantanal region are normal. Therefore, the use of these values as a baseline for this species would allow evaluating the effect of different captive management systems on the GC secretion and, consequently, the appearance of stress.

In general, captive animals do not have to deal with the stress-inducing factors that normally exist in natural environments such as predation, lack of food, or adverse weather conditions. This more protective environment could help animals to keep low GC profiles such as those observed in all captive parrots used in this report independently of the husbandry system. These results are in agreement with those reported in Siberian tigers, where the exposure to severe environmental conditions resulted in a significantly higher HPA axis activity in free-living tigers compared with captive tigers, more sheltered by the zoo facilities (Naidenko *et al.*, 2011). However, captivity by itself is a limiting factor for animals, and in some occasions, they are unable to adapt with a consequent increase in their GC levels, as reported in a study comparing faecal cortisol metabolites levels between captive and wild cheetah (*Acinonyx jubatus*) (Terio *et al.*, 2004). In such case, however, the poor genetic variability of this species is suggested as one of the reasons for chronic stress in these animals. Unlike cheetahs, the Blue-fronted amazon has a high genetic diversity (Caparroz *et al.*, 2000), allowing its populations to adapt better to challenging environments.

Acclimatisation appears to be an important aspect of stress in captivity. Unacclimated wild-caught sparrows also presented higher corticosterone values in comparison to their wild, free-living counterparts (Marra *et al.*, 1995). For Gray wolves (*Canis lupus*), corticosteroid-induced alkaline phosphatase activity, an isoenzyme commonly used to quantify stress in canids (Ochi *et al.*, 2013), was detected in some of the free-ranging wolves but not in long-term captive animals (Constable *et al.*, 1998). In our study, all the captive parrots were under captivity for years before the faecal sample collection, having already been habituated to the confinement conditions and, consequently, showing a lower stress response. Although chronic stress has also been associated with low GC levels in birds (Rich and Romero, 2005; Cyr and Romero, 2007) and could account for our findings with captive parrots, this is unlikely to be the case, especially for the favourable reproductive outcomes obtained by the breeding animals, suggestive of absence of chronic stress (Möstl and Palme, 2002). Moreover, it is well known that chronic stress plays a very important role in the development of behaviour disorders in captive parrots, such as the feather damaging behaviour (van Zeeland *et al.*, 2009; Ferreira *et al.*, 2015; Costa *et al.*, 2016). This abnormal behaviour is commonly used to measure welfare in both production and zoo animals (Dixon, 2008), and it is also very frequently observed in Psittacidae species (see review Van Zeeland *et al.*, 2013), including *Amazona* spp. parrots (Garner *et al.*, 2006), maintained in suboptimal environment conditions (Costa *et al.*, 2016). We can thus infer that the low GC levels in these animals may not be attributed to chronic stress but to the good captive conditions provided to the animals in all husbandry systems studied. Although not statistically significant, parrots from private houses, which probably received more attention and loving care by the owners, showed the lowest levels of uGCM, supporting the idea of good living conditions provided to these animals. In this context, the parrots kept as pets frequently share a similar life story, being illegally captured in the wild as chicks and hand-rearing to encourage imprinting. In this way, the owners manage to maintain a close and loving relationship with their companion animals (Low, 1985). Hundreds of parrot chicks are annually seized from the illegal wildlife trade in Brazil and sent to rehabilitation centres where they receive care until they are ready to fly and return to the wild, preferentially (Santos and Lopes, 2006). However, the indiscriminate release of hundreds of hand-reared animals can result in serious problems for natural populations such as diseases (Raso *et al.*, 2004; Ecco *et al.*, 2009; Godoy and Matushima, 2010; Saidenberg *et al.*, 2012), behavioural disorders (Chapple *et al.*, 2012) or exogamic depression (Banes *et al.*, 2016). In fact, during our sample collection, we witnessed the release of a captive group of Blue-fronted parrots in the Fazenda São Francisco area, which showed behavioural disorders a few months later. The sudden change of habitat could have lead them to an allostatic overload of their regulatory systems, preventing the development of an adequate adrenocortical response by the HPA axis (Matos *et al.*, 2017) and causing a maladaptation of these animals in the wild (Santos and Lopes, 2006). This unsatisfactory stress response has been already further underlined by some studies that associated low GCM levels in domesticated or captive animals with poor welfare or disease (Dorsey *et al.*, 2010; Pawluski *et al.*, 2017). Therefore, in a situation where captive Blue-fronted parrots show no signs of chronic stress, the real biological value of their release in nature is questionable. Once adapted to captivity, these individuals might be better suited to other purposes, such as environmental education in zoos, or as breeding animals in registered breeders to produce parrots destined to the pet legal market (Alves *et al.*, 2013).

In conclusion, the biological validation carried out in this study shows that this corticosterone EIA is capable of monitoring biologically relevant changes in uGCM in the captive and free-living Blue-fronted parrots*.* To our knowledge, this is the first study to measure stress hormone levels in a natural population of this species. The higher uGCM levels obtained in the wild population point to an adaptive response for their survival and the species propagation in a more challenging environment, in comparison to captive animals with lower concentrations. This study showed how appropriate captive conditions may contribute to reducing uGCM levels in parrots. However, low GC concentrations cannot be considered *per se* as a welfare indicator, but they must be complemented with the determination of other parameters such as breeding success and/or the absence of abnormal behaviours, frequently observed in this group of birds. The results obtained from captive parrots should help to spread a more adequate idea about captivity, showing that it is not a stressful habitat for wildlife despite being frequently labelled as such. A good understanding about the biology of the species, including the ‘basal’ levels of the stress hormones in captive and free-living animals, could also help to prevent the development of chronic stress or to apply corrective measures to improve the welfare and conservation both *in situ* and *ex-situ*. Overall, we hope to highlight the high capacity of the Blue-fronted amazon parrot to adapt to captivity conditions and its potential as a legalized pet, thus encouraging a higher presence of this species in private houses and in registered breeders. As the pet market grows, legal reproduction of Psittaciformes in captivity could become an important tool for the conservation of natural populations. However, professional breeding and further research to improve the reproductive outcomes of this species in captivity are extremely necessary.
